# Cases of Tardy Fecundity

**Published:** 1843-06

**Authors:** Daniel Stahl

**Affiliations:** Vincennes, Indiana


ART. III.—*Cases of Tardy Fecundity.*—Translated from a French manuscript* for the Western Journal of Medicine and Surgery. By DANIEL STAHL, M. D., of Vincennes, Indiana.

Fecundity of a man 99 years of age. John Moulard of Berri, died in 1710, at the age of 110 years, almost sound in feeling and judgment. He had married 10 wives, the last at the age of 99 years, she being 18 years old. Two years after she had a son.—*Almanach des Centenaires* of the year 1761, p. 67.

of a man at 100. A peasant died at Sinkoping, in Sweden, at the

* I received this manuscript from a highly valued and now departed friend, who, while pursuing his medical studies at Paris, collected the above cases. Exchanging subsequently the ministry of the body for that of the soul, I feel some delicacy in giving his name to the public, which moreover is the less necessary, as the reader, in most of the cases related, can refer to the sources whence hey have been derived.

age of 108 years; was married to the third wife at the age of 100 and had a son from that marriage.—*Journal de Verdun*, 1754, p. 103.

Fecundity of a man 92 years of age. Antoine Nonathac, in Limausin, died in 1757, at the age of 115 years; married three times; the second time at the age of 92, from which marriage he left some children; and the third time at 102 years.—*Affiches de Paris*, 1758, page 191; *Gazette de France*, 1758, page 139.

of a man at 86. Massinissa, king of Numidia, had at 86 years of age, a son born to him, named Metimnatus.—*Valer. Maxim.*, book 8, ch. 7.

of a woman at 59. Mary Volmerange died at Metz, at the age of 100 years, in 1742, had 24 children, the last of which she had at the age of 59.—*Etrenne Mignon de* 1743.

of a man at 91. Chevalier Bulstrade died at St. Germain, in 1711, at the age of 105; had 17 children, the last of which was born to him in his 91st year.—*Almanach des Centenaires*, 1761, p. 40.

of a woman at 106. A woman of Carpentras, still had her catamenia at the age of 106 years.—*Memoirs of the Academy of Sciences.*

of a man at 105. The Journal Encyclopedique of the year 1779, relates the case of a man then living at Tau, who at the age of 110 was very active (trés agile). He married at the age of 105 a young girl, who two years afterwards had a child by him.

of a man at 88. In 1762, near Varlovie, at the place of Mr. Zaluski, Staroste of Goujeck, there died a peasant at the age of 157 years. At the age of 88 (after having lived 58 years with his first wife) he married again and had 7 children.

of a man at 82. Jean Rovin died in Hungary, in 1740, at the age of 172 years; his wife was 164 years old. They lived together in wedlock 147 years. At the birth of their youngest son, the father was 82, and

Fecundity of a woman at 74. the mother 74 years old.—*Mercure de France*, 1756, p. 157; *Journal de Verdun*, 1740, p. 299; *Alman. des Centenaires*, 1761, p. 122.

of a man at 88 years of age. Pierre Zorten, of Hungary, died in 1724, at 185 years of age; had a son born to him at 88.—*Journal de Verdun*, 1740, p 299; *Mercure*, 1756, p. 157.

of a man of 80. A man 80 years old had his wife pregnant with the 6th child.—*Hobuken Epist.* 13.

of a man at 110 and one at 112 years. Two brothers cited by Buffon.

of a man at 70. This man gave no manifestations of puberty until be was 50 years of age; he was married at 70 and had 5 children, and died at the age of 120. *Buffon*, tom. 8, p. 132.

of a man at 90. A Spanish surgeon in the diocese of Comminges was married to his second wife at the age of 90 years, and became father of a very vigorous daughter (une fille trés vigoureuse). Died at 112 years.—*Gazette de France*, 1759, p. 60; *Affiches de Paris*, 1759, p. 12.

of a woman at 69. A woman of 69 years menstruated, but was sickly.

of a woman at 79. A nun menstruated at 79 years of age.

of a woman at 80. Two women of more than 80 years menstruated. Cited by Louis Courseur.—*Enc. Meth. Medicin.* tom. 2, p. 506.

of a woman at 67. Valesins has seen a woman having a child at 61 and continue to have them to the age of 67.

of a woman at 70. A woman in Avignon had a son at 70 years.—*Paré*, in folio, p. 623.

of a woman at 83. In the diocese of Seeze, a man of 94 years, married a woman of 83, and they had a son together. The Bishop of the Diocese, who knew them, cited the fact to the Academy of Sciences, and it is reported by his historian.—*Merveilles de la Nature*, art. *Fecundité*.

Fecundity of a man at 95, and a woman at 65. In the Journal Politique, of 1773, it is stated that the wife of Sieur Grant of Newri, aged 65 years, was delivered of a son; and to heighten the marvel of this circumstance, it is stated that the father, Sieur Grant, was 95 years of age.

of a woman at 60. A woman of 60 years was delivered of two sons (twins) at Sally, in Hungary.—*Extrait des Annales de la Republ. Francois, Journal du 27 Vendemaire,* an 7.

of a woman of 60. (Goulard has given the details.) A noble Venitian lady, was delivered at the age of 60, after a pregnancy of 15 months. (Two facts very apocryphal.)—*Nouvelles Observations sur les naissances tardives.* Paris, 1765, p. 95.

of a woman at 60, and a man at 70. *Goulard.* Another noble Venitian lady, aged 60 years, had a daughter by her husband, aged 70.—*Musa Epist.* 29, *t.* 2.

of a man at 80. Cato, at the age of 80, impregnated the daughter of one of his clients.

of a man at 100. Nicholaus, of Polavicene, had a child born to him at the age of 100 years.—*Ibid.*

of a man at 100. Leninius cites the fact of a man aged 100 years, who married at Stockholm a woman of 30, and had several children by her.—*Ibid.* p. 96.

of a man at 100. Platerus, Liv. 1. observes that his great father on his mother's side, aged more than 100 years, married a girl of 30, and had a daughter; and of a man at 77. that his father, Thomas Platerus, married at 77 years of age, and had 6 children.
*Ibid.* p. 96.

of a man at 73. Segismund Polastre, a physician, who taught Philosophy at Padua, having had the misfortune to loose his 4 sons, married again at the age of 70, and had yet 3 sons. He died at the age of 94 years.—*Recueil d'Histoires de Simon Goulard.*

of a woman at 54. The Countess of Fiasque, had, at the age of 54, a daughter by her husband older than herself.—*Ibid.*

Fecundity of a woman at 67.

A woman who menstruated yet at 60 years of age, had 3 children after the age of 67 years. *Ibid.*

of a woman of 68, and a man of 78.

"The Countess of Chaumerai, aged 68 years, was delivered, on the twenty-ninth of November, 1797, of a hardy son, at Castillon, Department de la Dordogne; the father is 78 years old."—*Extracted from public papers of that date.*

